# FOSL1 modulates Schwann cell responses in the wound microenvironment and regulates peripheral nerve regeneration

**DOI:** 10.1016/j.jbc.2023.105444

**Published:** 2023-11-08

**Authors:** Qianqian Chen, Lan Zhang, Fuchao Zhang, Sheng Yi

**Affiliations:** Key Laboratory of Neuroregeneration of Jiangsu and Ministry of Education, Co-innovation Center of Neuroregeneration, NMPA Key Laboratory for Research and Evaluation of Tissue Engineering Technology Products, Nantong University, Nantong, Jiangsu, China

**Keywords:** FOSL1, transcription factor, Schwann cell proliferation, Schwann cell migration, wound microenvironment, peripheral nerve regeneration

## Abstract

Peripheral glial Schwann cells switch to a repair state after nerve injury, proliferate to supply lost cell population, migrate to form regeneration tracks, and contribute to the generation of a permissive microenvironment for nerve regeneration. Exploring essential regulators of the repair responses of Schwann cells may benefit the clinical treatment for peripheral nerve injury. In the present study, we find that FOSL1, a AP-1 member that encodes transcription factor FOS Like 1, is highly expressed at the injured sites following peripheral nerve crush. Interfering FOSL1 decreases the proliferation rate and migration ability of Schwann cells, leading to impaired nerve regeneration. Mechanism investigations demonstrate that FOSL1 regulates Schwann cell proliferation and migration by directly binding to the promoter of EPH Receptor B2 (EPHB2) and promoting EPHB2 transcription. Collectively, our findings reveal the essential roles of FOSL1 in regulating the activation of Schwann cells and indicate that FOSL1 can be targeted as a novel therapeutic approach to orchestrate the regeneration and functional recovery of injured peripheral nerves.

Peripheral nerve injury remains a universal clinical issue with high morbidity, disability, and economic burden (1, 2). Cellular and molecular investigations demonstrate that damage to peripheral nerves induces drastic wound responses, including Wallerian degeneration, influx of inflammatory cells, and activation of Schwann cells (3, 4). Schwann cells are distinctive glial cells in the peripheral nervous system. Following peripheral nerve injury, Schwann cells in the wound microenvironment convert from a mature state to a repair phenotype with high proliferation and migration abilities (5, 6). Modulating the phenotype switch of Schwann cells influences the regeneration microenvironment and hence regulates the regeneration process (7).

Transcription factors are master regulators of a variety of biological processes, ranging from cellular activity to organ development and tissue regeneration (8, 9). Transcription factors bind to DNA in a sequence-specific manner, control gene transcription, and regulate various physiological and pathological events (9). The striking abilities of transcription factors in inducing the dedifferentiation and transdifferentiation of somatic cells imply that transcription factors may mediate the reprogramming of Schwann cells and the regeneration of injured peripheral nerves (10, 11, 12). It has been demonstrated that the phenotype modulation of Schwann cells can be regulated by many transcriptional factors, such as c-Jun, Sox2, and Stat3 (13). For instance, the absence of c-Jun abolishes injury-elicited molecular changes in Schwann cells and leads to abnormal regeneration tracks as well as impaired function recovery (14).

High-throughput analyses make the determination of the dynamic expression patterns of transcription factor-coding genes after peripheral never injury available and accordingly contribute to the cognition of many essential transcriptional factors for nerve regeneration. Our previous microarray analyses of injured rat sciatic nerves demonstrate that FOSL1, a gene that encodes AP-1 family member transcription factor FOS Like 1, is upregulated in distal nerve stumps after nerve injury (15). Compared with distal nerve stumps, Schwann cells in the wound region of injured nerves undergo more robust reprogramming responses (6). Whether FOSL1 exhibits altered expression at the injured sites and more importantly, the biological functions of FOSL1 in nerve regeneration remains to be examined. Herein, we explore the expression changes of transcription factor FOSL1 at the injured sites after rat sciatic nerve crush, determine the effects of FOSL1 on Schwann cell activation and peripheral nerve regeneration, and identify EPH Receptor B2 (EPHB2) as a downstream target of FOSL1.

## Results

### FOSL1 is upregulated at the injured sites following sciatic nerve injury

**Figure 1:**
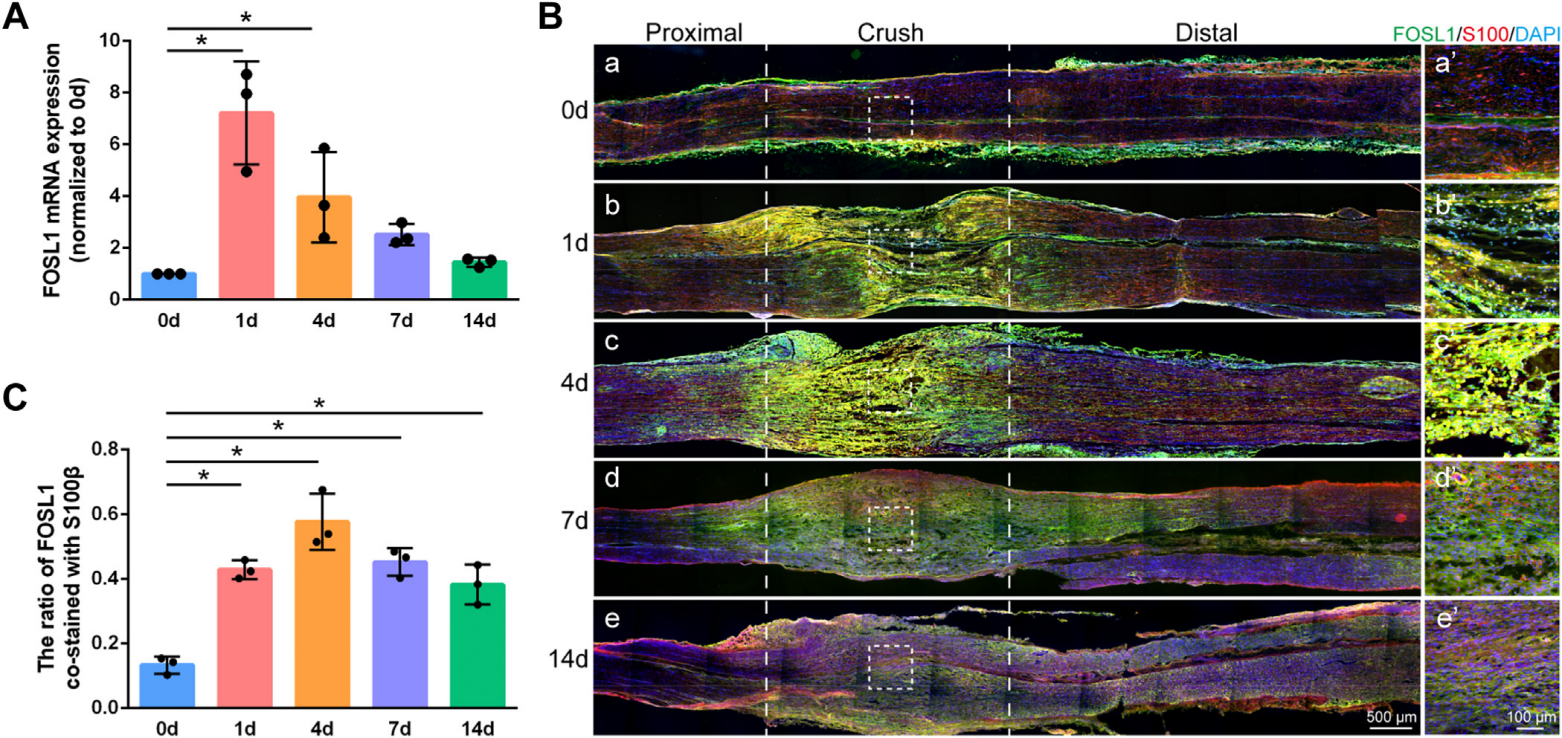
**Expressions of FOSL1 in rat sciatic nerves following nerve crush injury.***A*, relative mRNA expressions of FOSL1 in rat sciatic nerves at 0, 1, 4, 7, and 14 days post nerve injury. Summarized data are presented as mean ± S.D. (n = 3 independent biological replicates; ∗adjusted *p*-value < 0.05 *versus* 0-days control, ordinary one-way ANOVA followed by Dunnett’s multiple comparisons test; adjusted *p*-value < 0.001 for 1 day *versus* 0 days, adjusted *p*-value = 0.041 for 4 days *versus* 0 days, adjusted *p*-value = 0.390 for 7 days *versus* 0 days, and adjusted *p*-value = 0.971 for 14 days *versus* 0 days). *B*, representative immunohistochemistry images of rat sciatic nerves at 0, 1, 4, 7, and 14 days post nerve injury. *Green* represents FOSL1 staining, *red* represents S100β staining, and *blue* represents nuclear staining. The injured sites are labeled with *dashed lines*. *Boxed areas* are enlarged on the *right*. Scale bar: *left*, 500 μm; *right*, 100 μm. *C*, quantification of the co-occurrence of FOSL1 and S100β at the injured sites. Summarized data are presented as mean ± SD (n = 3 independent biological replicates; ∗adjusted *p*-value < 0.05 *versus* 0-days control, ordinary one-way ANOVA followed by Dunnett’s multiple comparisons test; adjusted *p*-value < 0.001 for 1 day *versus* 0 days, adjusted *p*-value < 0.001 for 4 days *versus* 0 days, adjusted *p*-value < 0.001 for 7 days *versus* 0 days, and adjusted *p*-value < 0.001 for 14 days *versus* 0 days).

To evaluate the expression patterns of FOSL1 at the injured sites following sciatic nerve injury, real-time RT-PCR was conducted and the relative abundances of FOSL1 at 1, 4, 7, and 14 days post nerve injury were calculated and normalized to uninjured 0-days control. Gene quantification results showed that FOSL1 was immediately up-regulated at early time points post nerve injury. At 1 day after nerve injury, FOSL1 mRNA abundance exhibited a more than 7-fold up-regulation as compared with its expression in uninjured 0-day control. FOSL1 mRNA was kept expressed at relatively high levels at later time points, with an approximately 4-fold upregulation at 4 days after nerve injury and an approximately 2.5-fold upregulation at 7 days after nerve injury (Fig. 1*A*). Immunohistochemistry of sciatic nerves showed elevated FOSL1 protein expressions at the injured sites (Fig. 1*B*). Sciatic nerves were further co-immunostained with FOSL1 and Schwann cell marker S100β to quantify FOSL1 and S100β double-positive signals. The high co-occurrence of FOSL1 and S100β after nerve injury showed that elevated FOSL1 after nerve injury may largely be expressed by Schwann cells (Fig. 1*C*).

### FOSL1 modulates Schwann cell behavior

**Figure 2:**
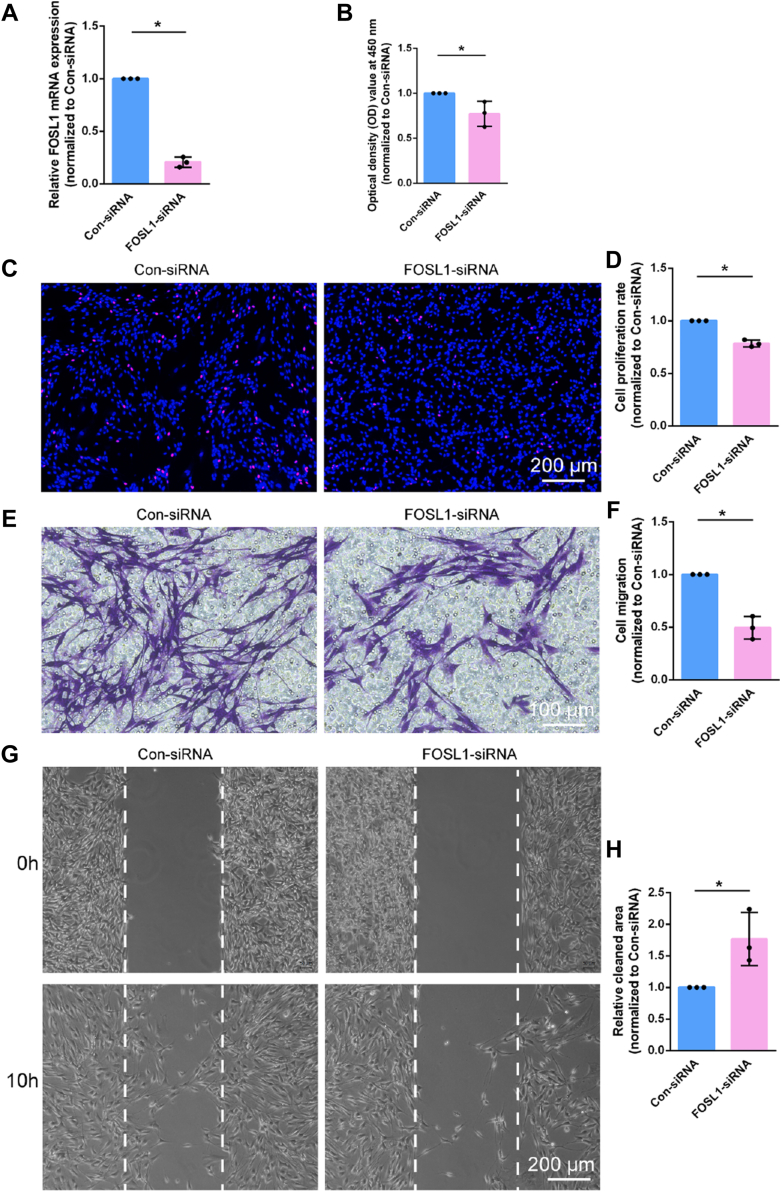
**FOSL1 knockdown regulates Schwann cell phenotype.***A*, relative mRNA expressions of FOSL1 in Schwann cells transfected with FOSL1 siRNA or control siRNA. Summarized data are presented as mean ± S.D. (n = 3 independent biological replicates; ∗*p*-value < 0.05, unpaired *t* test; *p*-value < 0.001). *B*, quantification of the optical density values of Schwann cells transfected with FOSL1 siRNA or control siRNA. Summarized data are presented as mean ± S.D. (n = 3 independent biological replicates; ∗*p*-value < 0.05, unpaired *t* test; *p*-value = 0.047). *C*, representative EdU staining images of Schwann cells transfected with FOSL1 siRNA or control siRNA. *Red* represents EdU staining and *blue* represents nuclear staining. Scale bar: 200 μm. *D*, quantification of the proliferation rate of Schwann cells after transfection with FOSL1 siRNA or control siRNA. Summarized data are presented as mean ± S.D. (n = 3 independent biological replicates; ∗*p*-value < 0.05, unpaired *t* test; *p*-value < 0.001). *E*, representative transwell migration images of Schwann cells transfected with FOSL1 siRNA or control siRNA. *Purple* represents migrated Schwann cells. Scale bar: 100 μm. *F*, quantification of the migration ability of Schwann cells after transfection with FOSL1 siRNA or control siRNA. Summarized data are presented as mean ± S.D. (n = 3 independent biological replicates; ∗*p*-value < 0.05, unpaired *t* test; *p*-value = 0.001). *G*, representative wound healing images of Schwann cells transfected with FOSL1 siRNA or control siRNA at 0 and 10 h after insert removal. Insert positions are labeled with *dashed lines*. Scale bar: 200 μm. *H*, quantification of the relative cleaned area at 10 h after insert removal in Schwann cells transfected with FOSL1 siRNA or control siRNA. Summarized data are presented as mean ± S.D. (n = 3 independent biological replicates; ∗*p*-value < 0.05, unpaired *t* test; *p*-value = 0.034).

To investigate the effects of FOSL1 on Schwann cells, a specific siRNA segment against FOSL1 was transfected into primary cultured Schwann cells. FOSL1 siRNA suppressed the mRNA expression of FOSL1 to approximately 20%, showing high knockdown efficiency (Fig. 2*A*). Outcomes from CCK-8 assay showed that Schwann cells transfected with FOSL1 siRNA had a significantly reduced optical density value to about 77.16% of Schwann cells transfected with control siRNA (Fig. 2*B*). Consistently, in FOSL1 siRNA-transfected Schwann cells, the percentage of EdU-positive cells in total cells was reduced to about 78.48% of Schwann cells transfected with control siRNA (Fig. 2, *C* and *D*). These observations indicate that FOSL1 knockdown results in suppressed Schwann cell proliferation.

Transwell migration assay demonstrated that after interfering with FOSL1 expression, the amount of migrated Schwann cells reduced to half of Schwann cells transfected with control siRNA (Fig. 2, *E* and *F*). Besides the longitudinal migration across transwell chambers, the transverse migration of transfected Schwann cells was determined according to the wound healing assay. In Schwann cells transfected with FOSL1 siRNA, considerably less migrating cells were observed in the wound area and a larger cleaned area was left at 10 h after wounding (Fig. 2, *G* and *H*). These findings fully indicate that FOSL1 knockdown leads to impaired Schwann cell migration.

**Figure 3:**
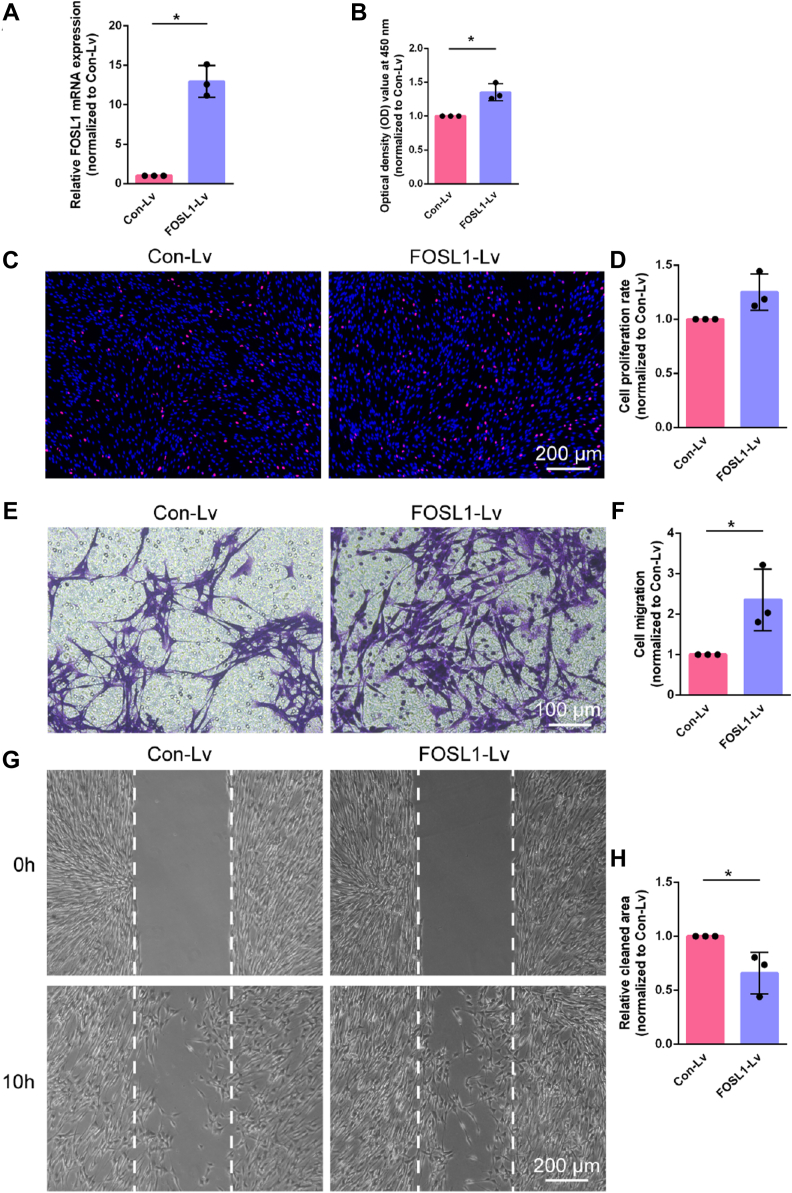
**FOSL1 overexpression regulates Schwann cell phenotype.***A*, relative mRNA expressions of FOSL1 in Schwann cells infected with FOSL1-overexpressing lentivirus or control lentivirus. Summarized data are presented as mean ± S.D. (n = 3 independent biological replicates; ∗*p*-value < 0.05, unpaired *t* test; *p*-value = 0.009). *B*, quantification of the optical density values of Schwann cells transfected with FOSL1-overexpressing lentivirus or control lentivirus. Summarized data are presented as mean ± S.D. (n = 3 independent biological replicates; ∗*p*-value < 0.05, unpaired *t* test; *p*-value = 0.008). *C*, representative EdU staining images of Schwann cells transfected with FOSL1-overexpressing lentivirus or control lentivirus. *Red* represents EdU staining and *blue* represents nuclear staining. Scale bar: 200 μm. *D*, quantification of the proliferation rate of Schwann cells after transfection with FOSL1-overexpressing lentivirus or control lentivirus. Summarized data are presented as mean ± S.D. (n = 3 independent biological replicates; *p*-value = 0.062). *E*, representative transwell migration images of Schwann cells transfected with FOSL1-overexpressing lentivirus or control lentivirus. *Purple* represents migrated Schwann cells. Scale bar: 100 μm. *F*, quantification of the migration ability of Schwann cells after transfection with FOSL1-overexpressing lentivirus or control lentivirus. Summarized data are presented as mean ± SD (n = 3 independent biological replicates; ∗*p*-value < 0.05, unpaired *t* test; *p*-value = 0.037). *G*, representative wound healing images of Schwann cells transfected with FOSL1-overexpressing lentivirus or control lentivirus at 0 and 10 h after insert removal. Insert positions are labeled with *dashed lines*. Scale bar: 200 μm. *H*, quantification of relative cleaned area at 10 h after insert removal in Schwann cells transfected with FOSL1-overexpressing lentivirus or control lentivirus. Summarized data are presented as mean ± S.D. (n = 3 independent biological replicates; ∗*p*-value < 0.05, unpaired *t* test; *p*-value = 0.038).

Schwann cells were further transfected with FOSL1-overexpressing lentivirus to examine the functional roles of elevated FOSL1 in Schwann cells (Fig. 3*A*). Following FOSL1-overexpressing lentivirus transfection, the optical density value showed a 1.35-fold increase (Fig. 3*B*) and the percentage of EdU-positive cells showed a 1.25-fold increase (Fig. 3, *C* and *D*). Enhanced cell migration capacity was also detected in FOSL1-overexpressing lentivirus-transfected Schwann cells based on an increased amount of cells crossed transwell chambers in the transwell migration assay (Fig. 3, *E* and *F*) and a decreased cleaned area in the wound healing assay (Fig. 3, *G* and *H*). These findings demonstrate that in contrast to FOSL1 silencing, elevated FOSL1 supports Schwann cell proliferation and migration.

### FOSL1 influences peripheral nerve regeneration

**Figure 4:**
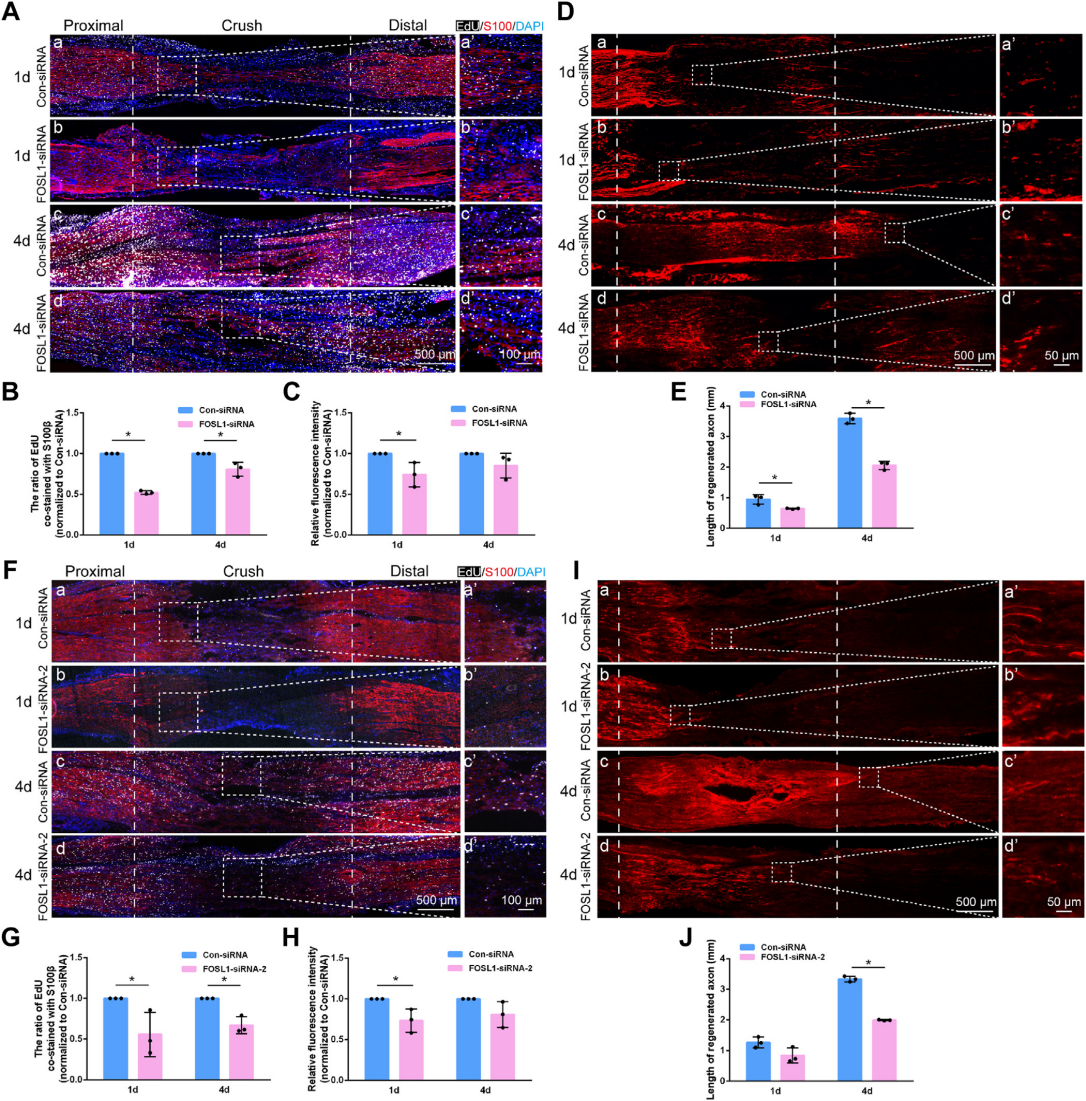
**FOSL1 knockdown impairs Schwann cell activity and axon elongation after nerve injury.***A*, representative EdU and S100β staining in rat sciatic nerves at 1 and 4 days after nerve injury and siRNA (FOSL-siRNA) injection. *White* represents EdU staining, *red* represents S100β staining, and *blue* represents nuclear staining. The injured sites are labeled with *dashed lines*. *Boxed areas* are enlarged on the *right*. Scale bar: *left*, 500 μm; *right*, 100 μm. *B*, quantification of EdU and S100β co-occurrence at the injured sites. Summarized data are presented as mean ± S.D. (n = 3 independent biological replicates; ∗*p*-value < 0.05, unpaired *t* test; *p*-value < 0.001 at 1 day and *p*-value = 0.018 at 4 days). *C*, quantification of mean fluorescence intensities of S100β at the injured sites. S100β fluorescence intensity was measured at the same distance from the injured sites in rats injected with FOSL1 siRNA or control siRNA. Summarized data are presented as mean ± S.D. (n = 3 independent biological replicates; ∗*p*-value < 0.05, unpaired *t* test; *p*-value =0.041 at 1 day and *p*-value = 0.167 at 4 days). *D*, representative SCG10 staining in rat sciatic nerves at 1 and 4 days nerve injury and siRNA (FOSL-siRNA) injection. *Boxed areas* are enlarged on the *right*. *Red* represents SCG10 staining. Scale bar: *left*, 500 μm; *right*, 50 μm. *E*, quantification of length of regenerated nerve fiber. Summarized data are presented as mean ± SD (n = 3 independent biological replicates; ∗*p*-value < 0.05, unpaired *t* test; *p*-value = 0.027 at 1 day and *p*-value < 0.001 at 4 days). *F*, representative EdU and S100β staining in rat sciatic nerves at 1 and 4 days after nerve injury and siRNA (FOSL-siRNA-2) injection. *White* represents EdU staining, *red* represents S100β staining, and *blue* represents nuclear staining. The injured sites are labeled with *dashed lines*. *Boxed areas* are enlarged on the *right*. Scale bar: *left*, 500 μm; *right*, 100 μm. *G*, quantification of EdU and S100β co-occurrence at the injured sites. Summarized data are presented as mean ± SD (n = 3 independent biological replicates; ∗*p*-value < 0.05, unpaired *t* test; *p*-value = 0.047 at 1 day and *p*-value = 0.006 at 4 days). *H*, quantification of mean fluorescence intensities of S100β at the injured sites. S100β fluorescence intensity was measured at the same distance from injured site in rats injected with FOSL1 siRNA or control siRNA. Summarized data are presented as mean ± SD (n = 3 independent biological replicates; ∗*p*-value < 0.05, unpaired *t* test; *p*-value = 0.033 at 1 day and *p*-value = 0.102 at 4 days). *I*, representative SCG10 staining in rat sciatic nerves at 1 and 4 days after nerve injury and siRNA (FOSL-siRNA-2) injection. *Boxed areas* are enlarged on the *right*. *Red* represents SCG10 staining. Scale bar: *left*, 500 μm; *right*, 50 μm. *J*, quantification of the length of the regenerated nerve fiber. Summarized data are presented as mean ± S.D. (n = 3 independent biological replicates; ∗*p*-value < 0.05, unpaired *t* test; *p*-value = 0.072 at 1 day and *p*-value < 0.001 at 4 days).

*In vivo* effect of FOSL1 siRNA was explored by directly injecting FOSL1 siRNA into rats that underwent sciatic nerve crush injury. EdU staining revealed the existence of several proliferating cells in injured nerves of rats injected with FOSL siRNA or control siRNA at 1 day after nerve injury. Obviously, more proliferating cells could be detected at 4 days post surgery (Fig. 4*A*). The number of EdU-stained proliferating cells in FOSL siRNA-injected rats was noticeably smaller as compared with rats injected with control siRNA. Quantification of cells co-stained with EdU and S100β demonstrated that there existed less amount of proliferating Schwann cells after FOSL1 siRNA injection, suggesting the inhibiting role of FOSL1 siRNA in Schwann cell proliferation (Fig. 4, *A* and *B*). Immunostaining with S100β showed that Schwann cells migrated from both the proximal and the distal nerve segments toward the injured sites (Fig. 4*A*). Diminished fluorescence intensity of S100β was observed in FOSL1 siRNA-injected rats (Fig. 4, *A* and *C*), indicating that reduced FOSL1 expression suppresses Schwann cell migration. The growth conditions of axons were determined by immunostaining with axon marker SCG10. The comparison between FOSL1 siRNA and control siRNA-injected rats showed that FOSL1 siRNA injection considerably hinders the growth of SCG10-positive axons (Fig. 4*D*). In rats injected with control siRNA, axons regenerated for about 0.95 mm and 3.59 mm at 1 and 4 days after nerve injury, respectively. But the growth conditions of axons in rats injected with FOSL siRNA-injected rats were noticeably inferior to those injected with control siRNA (Fig. 4, *D* and *E*). Injection with another *in vivo* FOSL1 siRNA (FOSL1-siRNA-2) also resulted in reduced EdU-positive Schwann cells and S100β fluorescence intensity in the wound area as well as delayed axon regeneration from the proximal nerve stump, further demonstrating the inhibitory role of FOSL1 siRNA in peripheral nerve repair (Fig. 4, *F*–*J*).

**Figure 5:**
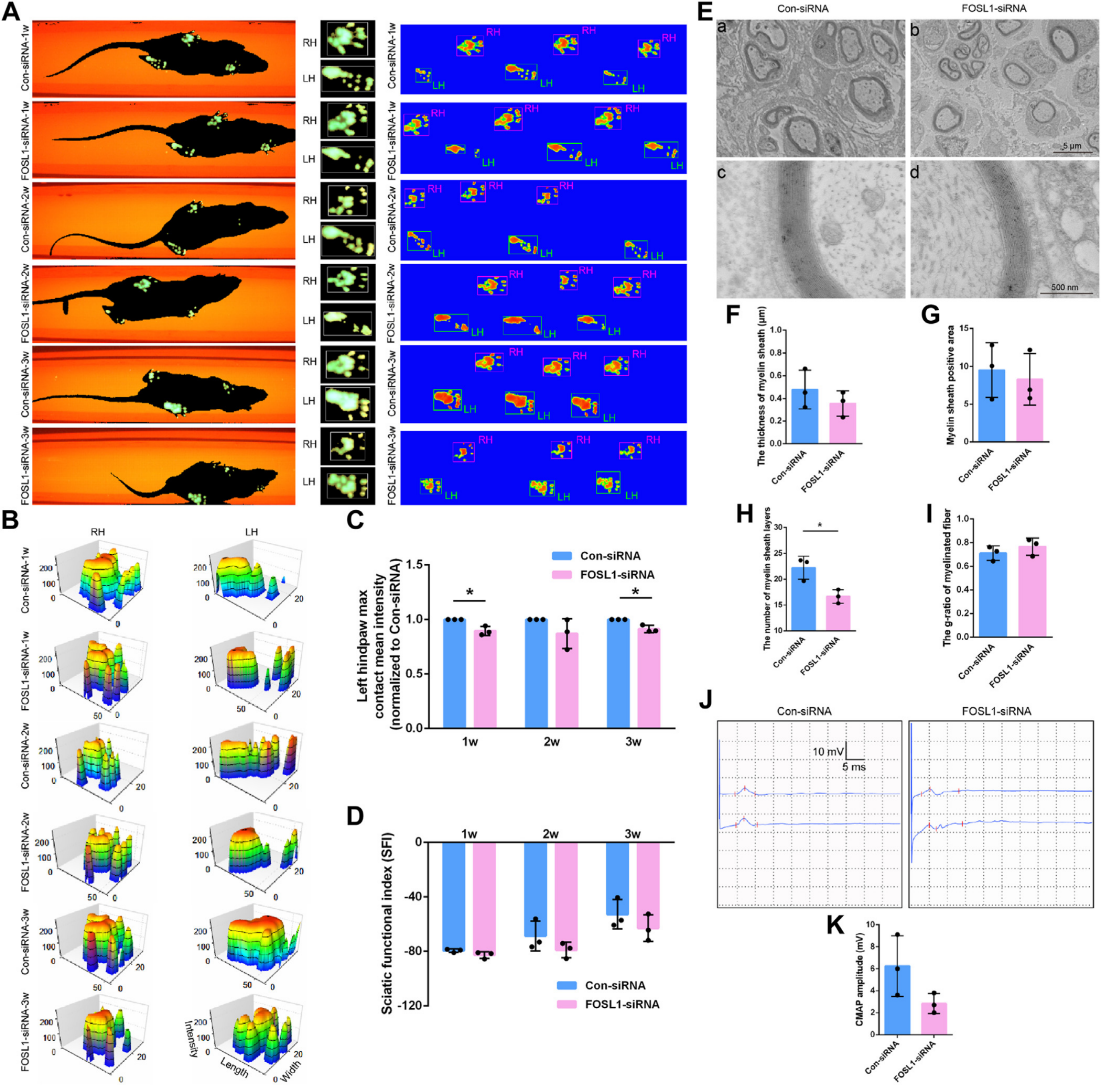
**FOSL1 knockdown hinders the functional recovery of peripheral nerve-injured rats.***A* and *B*, representative (*A*) footprint images and (*B*) 3D footprint intensities of rats injected with FOSL1 siRNA or control siRNA at 1, 2, and 3 weeks after surgery. RH represents an uninjured right hindpaw and LH represents an injured left hindpaw. *C*, quantification of left hind paw contact mean intensity. Summarized data are presented as mean ± S.D. (n = 3 independent biological replicates; ∗*p*-value < 0.05, unpaired *t* test; *p*-value = 0.012 at 1 week, *p*-value = 0.176 at 2 weeks, and *p*-value = 0.010 at 3 weeks). *D*, quantification of sciatic functional index (SFI). Summarized data are presented as mean ± S.D. (n = 3 independent biological replicates; *p*-value = 0.114 for 1 week, *p*-value = 0.221 for 2 weeks, and *p*-value = 0.293 for 3 weeks). *E*, representative transmission electron microscopy images of myelinated nerve fibers of rats injected with FOSL1 siRNA or control siRNA at 2 weeks after surgery. Scale bar: *upper*, 5 μm; *below*, 500 nm. *F*, quantification of the thickness of myelin sheath. Summarized data are presented as mean ± S.D. (n = 3 independent biological replicates; *p*-value = 0.357). *G*, quantification of myelin sheath positive area. Summarized data are presented as mean ± S.D. (n = 3 independent biological replicates; *p*-value = 0.695). *H*, quantification of the number of myelin sheath layers. Summarized data are presented as mean ± S.D. (n = 3 independent biological replicate; ∗*p*-value < 0.05, unpaired *t* test; *p*-value = 0.021). *I*, quantification of the g-ratio of myelinated fiber. Summarized data are presented as mean ± S.D. (n = 3 independent biological replicates; *p*-value = 0.363). *J*, representative CMAP recordings of rats injected with FOSL1 siRNA or control siRNA at 3 weeks after surgery. *K*, quantification of CMAP peak amplitude. Summarized data are presented as mean ± S.D. (n = 3 independent biological replicates; *p*-value = 0.112).

The effects of FOSL1 knockdown on the functional recovery of peripheral nerves were evaluated at longer time periods following nerve injury. The recovery of motor function was intuitively detected from the gaits of injured rats. It was observed that rats walked with a limp after sciatic nerve injury and their gaits were gradually recovering during regeneration. Compared with FOSL1 siRNA-injected rats, in rats injected with control siRNA, the differences between footprints of the uninjured right hind paw and injured left hind paw were smaller (Fig. 5, *A* and *B*). Measured 3D footprint intensities also showed that control siRNA-injected rats had relatively higher max contact mean intensities of injured left hind paws (Fig. 5, *B* and *C*). Calculated sciatic functional index (SFI) demonstrated SFI values of both control siRNA and FOSL1 siRNA-injected rats were about −80 at 1 week after surgery. At 2 weeks after surgery, FOSL1 siRNA-injected rats still had SFI values of nearly −80 while control-injected rats had increased SFI values of about −68.66. SFI values of control siRNA-injected rats remained slightly higher after 3 weeks (Fig. 5*D*). These observations implied that FOSL1 silencing induces delayed motor function recovery.

In addition to restricted hind paw movement, myelin sheathes in FOSL1 siRNA-injected rats were not well-developed and not tightly wrapped as compared with rats injected with control siRNA (Fig. 5*E*). FOSL1 siRNA-injected rats had relatively smaller nerve fiber myelin sheath thickness and myelin positive area (Fig. 5, *F* and *G*). The number of myelin sheath layers counted from transmission electron microscopy images with high magnifications was also found to be decreased after FOSL1 siRNA injection (Fig. 5*H*). Consistent with a decreased thickness of myelin sheath and reduced number of myelin sheath layers in FOSL1 siRNA-injected rats, the differences between the diameters of the inner axons and outer nerve fibers were smaller. Therefore, g-ratio, a ratio of inner axon diameter to outer nerve fiber diameter, slightly increased from an average value of 7.10 in the control siRNA-injected group to an average value of 0.77 in the FOSL1 siRNA-injected group (Fig. 5*I*). These observations demonstrated that reduced FOSL1 influences myelin sheathes surrounding axons and implied that reduced FOSL1 may negatively affect nerve conductance.

Compound muscle action potential (CMAP) was accordingly measured to determine responses to applied external electrical stimulus, aiming to disclose the recovery condition of nerve conductance. CMAP amplitude reduced to an untraceable level immediately after nerve injury and then progressively increased to a measurable level at 3 weeks post injury (Fig. 5*J*). A slightly lower amplitude was detected in rats injected with FOSL1 siRNA as compared with rats injected with control siRNA (Fig. 5*K*), indicating that FOSL1 knockdown suppresses the functional recovery of sciatic nerves from the electrophysiological aspect.

### FOSL1 affects Schwann cell activity *via* directly regulating EPHB2

**Figure 6:**
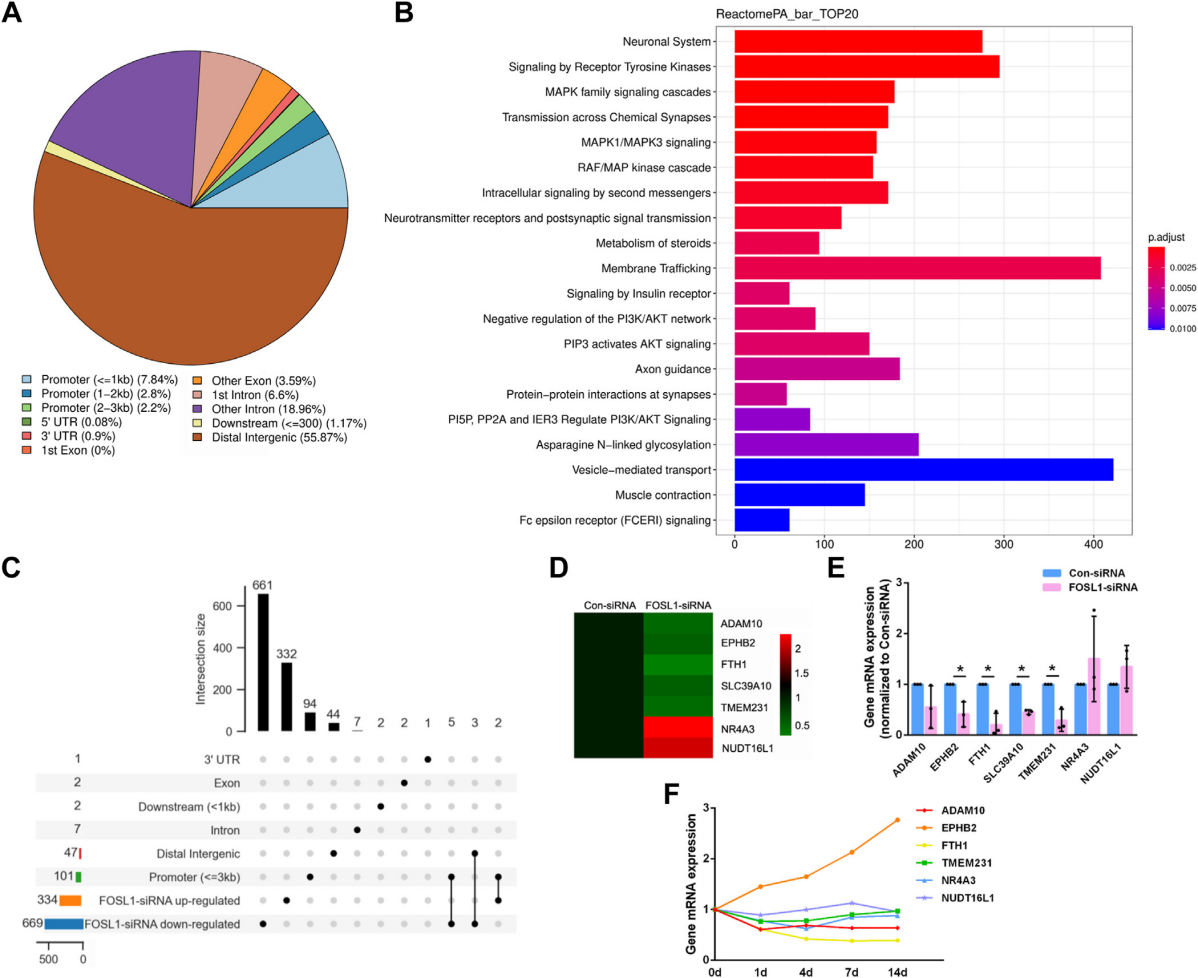
**Identification of FOSL1 bound chromatin-accessible regions and mediated gene changes.***A*, global distribution of FOSL1 bound elements. *B*, list of significantly enriched Reactome signaling pathways. *C*, an upset plot of distributions of FOSL1-regulated genes. The bar chart above represents the number of genes in each group. The bar chart at the *bottom left* represents the numbers of genes included in specific FOSL1 binding regions as well as up-regulated and down-regulated genes after FOSL1 siRNA transfection. The *dotted lines* at the *bottom right* represent grouping information. *D*, heatmap of RNA sequencing-determined expression patterns of ADAM10, EPHB2, FTH1, SLC39A10, TMEM231, NR4A3, and NUDT16L1 in Schwann cells after FOSL1 siRNA transfection. *Red* represents up-regulation and *green* represents down-regulation. *E*, relative mRNA expressions of ADAM10, EPHB2, FTH1, SLC39A10, TMEM231, NR4A3, and NUDT16L1 in Schwann cells after FOSL1 siRNA transfection. Summarized data are presented as mean ± SD (n = 3 independent biological replicates; ∗*p*-value < 0.05, unpaired *t* test; *p*-value = 0.350 for ADAM10, *p*-value = 0.015 for EPHB2, *p*-value = 0.004 for FTH1, *p*-value < 0.001 for SLC39A10, *p*-value = 0.005 for TMEM231, *p*-value = 0.358 for NR4A3, and *p*-value = 0.230 for NUDT16L1). *F*, RNA sequencing-determined expression trends of ADAM10, EPHB2, FTH1, SLC39A10, TMEM231, NR4A3, and NUDT16L1 in rat sciatic nerves at 0, 1, 4, 7, and 14 days post nerve injury.

To explore the potential targets of transcription factor FOSL1, chromatin immunoprecipitation (ChIP) sequencing was performed in Schwann cells infected with FOSL1-overexpressing lentivirus. A total of 104,901 FOSL1 consensus motif-containing peaks were identified, with roughly 12.84% elements concentrated at the proximal end (0–3 kb) to the transcription start sites, a region with high transcription activity (Fig. 6*A*) (16). Reactome pathway analysis revealed significantly enriched biological cascades in FOSL1-bound genes, including tyrosine kinase receptor signaling, MAPK signaling, and PI3K/AKT signaling (Fig. 6*B*). ChIP sequencing outcomes were jointly analyzed with RNA sequencing data of FOSL1 siRNA-transfected Schwann cells (Fig. 6*C*). Intersection of FOSL1 bound elements in the 0 to 3 kb promoter region and differentially expressed genes in FOSL1 siRNA-transfected Schwann cells uncovered a total of five downregulated genes, including ADAM10, EPHB2, FTH1, SLC39A10, and TEM231, as well as 2 upregulated genes NR4A3 and NUDT16L1. The expression levels of these identified genes were displayed in a heatmap according to sequencing results (Fig. 6*D*) and further validated using real-time RT-PCR (Fig. 6*E*). All genes showed expression trends that were consistent with sequencing outcomes, with EPHB2, FTH1, SLC39A10, and TMEM231 significantly decreased after FOSL1 siRNA transfection (Fig. 6*E*). The temporal expression patterns of these potential targets of FOSL1 were discovered using previous sequencing data of injured rat sciatic nerves (stored in SRA database with accession number PRJNA394957) (17, 18). Among these candidate target genes, EPHB2 was found to be robustly elevated in injured sciatic nerves, showing similar expression trends as FOSL1 (Fig. 6*F*). The positive correlation between EPHB2 and FOSL1 expression implied that EPHB2 may be a downstream target of FOSL1.

**Figure 7:**
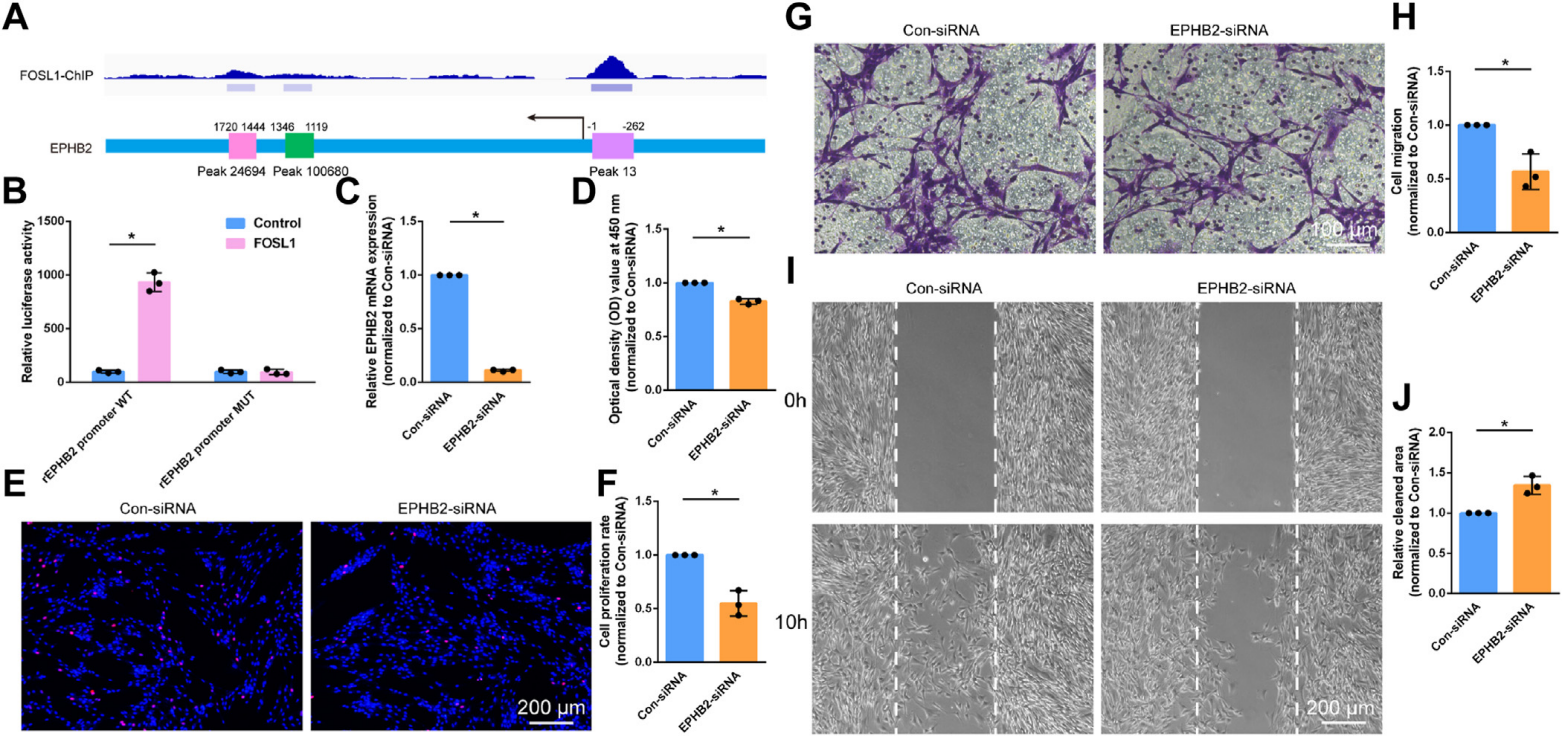
**FOSL1-regulated EPHB2 influences Schwann cell phenotype.***A*, genome view of FOSL1 binding on the promoter region of EPHB2. *B*, relative luciferase activity in cells co-transfected with wild-type or mutant EPHB2 promoter reporter vector and FOSL1 or control empty vector. Summarized data are presented as mean ± S.D. (n = 3 independent biological replicates; ∗*p*-value < 0.05, unpaired *t* test; *p*-value < 0.001 for wild-type promoter and *p*-value = 0.811 for mutant promoter). *C*, relative mRNA expressions of EPHB2 in Schwann cells transfected with EPHB2 siRNA or control siRNA. Summarized data are presented as mean ± S.D. (n = 3 independent biological replicates; ∗*p*-value < 0.05, unpaired *t* test; *p*-value < 0.001). *D*, quantification of the optical density values of Schwann cells transfected with EPHB2 siRNA or control siRNA. Summarized data are presented as mean ± S.D. (n = 3 independent biological replicates; ∗*p*-value < 0.05, unpaired *t* test; *p*-value < 0.001). *E*, representative EdU staining images of Schwann cells transfected with EPHB2 siRNA or control siRNA. *Red* represents EdU staining and *blue* represents nuclear staining. Scale bar: 200 μm. *F*, quantification of the proliferation rate of Schwann cells after transfection with EPHB2 siRNA or control siRNA. Summarized data are presented as mean ± SD (n = 3 independent biological replicates; ∗*p*-value < 0.05, unpaired *t* test; *p*-value = 0.003). *G*, representative transwell migration images of Schwann cells transfected with EPHB2 siRNA or control siRNA. *Purple* represents migrated Schwann cells. Scale bar: 100 μm. *H*, quantification of the migration ability of Schwann cells after transfection with EPHB2 siRNA or control siRNA. Summarized data are presented as mean ± S.D. (n = 3 independent biological replicates; ∗*p*-value < 0.05, unpaired *t* test; *p*-value = 0.010). *I*, representative wound healing images of Schwann cells transfected with EPHB2 siRNA or control siRNA at 0 and 10 h after insert removal. Insert positions are labeled with *dashed lines*. Scale bar: 200 μm. *J*, quantification of the relative cleaned area at 10 h after insert removal in Schwann cells transfected with EPHB2 siRNA or control siRNA. Summarized data are presented as mean ± SD (n = 3 independent biological replicates; ∗*p*-value < 0.05, unpaired *t* test; *p*-value = 0.006).

Next, luciferase reporter assay was conducted to investigate the transcription regulation of EPHB2. The genomic locus revealed 3 motif peaks on the promoter region of EPHB2, including peak 13 (−262 bp to −1 bp), peak 100,680 (1119 bp to 1346 bp), and peak 24,694 (1444 bp to 1720 bp), with peak 13 obtaining the highest peak value (Fig. 7*A*). Wild-type and mutant EPHB2 promoter constructs at peak 13 were constructed and co-transfected with FOSL1. Luciferase assay showed that the luciferase activity was significantly elevated after the co-transfection with FOSL1 and wild-type EPHB2 promoter but unchanged after the co-transfection with FOSL1 and mutant EPHB2 promoter (Fig. 7*B*). The biological functions of EPHB2 were then investigated by transfecting cultured primary Schwann cells with EPHB2 siRNA (Fig. 7*C*). Similar to FOSL1 silencing, interfering EPHB2 decreased the proliferation and migration of Schwann cells (Fig. 7, *D*–*J*).

**Figure 8:**
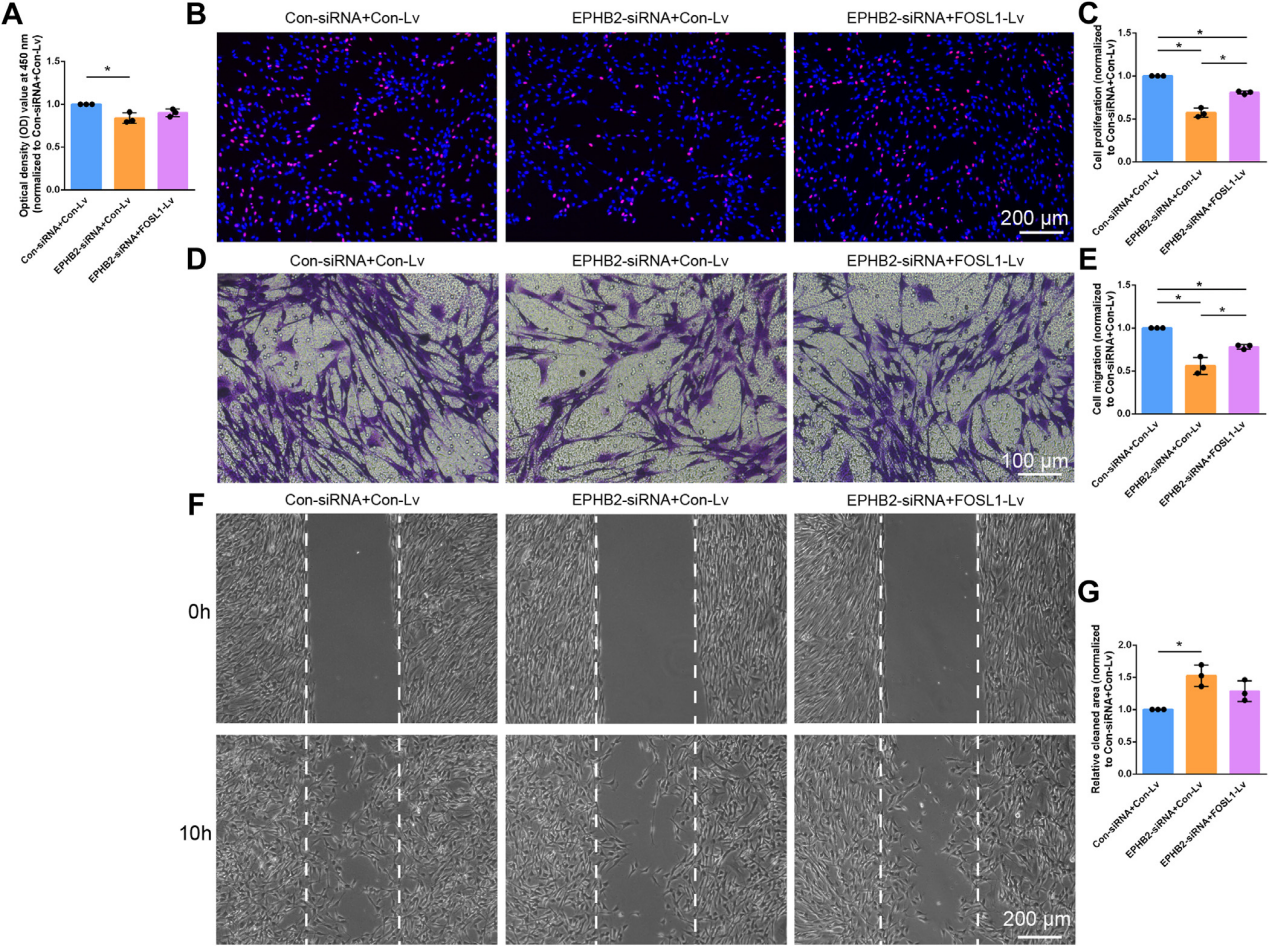
**FOSL1 overexpression partially rescues the inhibiting role of EPHB2 siRNA on Schwann cell behavior.***A*, quantification of the optical density values of Schwann cells co-transfected with control siRNA plus control lentivirus, EPHB2 siRNA plus control lentivirus, or EPHB2 siRNA plus FOSL1-overexpressing lentivirus. Summarized data are presented as mean ± SD (n = 3 independent biological replicates; ∗adjusted *p*-value < 0.05, ordinary one-way ANOVA followed by Sidak’s multiple comparisons test; adjusted *p*-value = 0.012 for EPHB2 siRNA plus control lentivirus *versus* control siRNA plus control lentivirus, adjusted *p*-value = 0.095 for EPHB2 siRNA plus FOSL1-overexpressing lentivirus *versus* control siRNA plus control lentivirus, and adjusted *p*-value = 0.343 for EPHB2 siRNA plus FOSL1-overexpressing lentivirus *versus* EPHB2 siRNA plus control lentivirus). *B*, representative EdU staining images of Schwann cells co-transfected with control siRNA plus control lentivirus, EPHB2 siRNA plus control lentivirus, or EPHB2 siRNA plus FOSL1-overexpressing lentivirus. *Red* represents EdU staining and *blue* represents nuclear staining. Scale bar: 200 μm. *C*, quantification of the proliferation rate of transfected Schwann cells. Summarized data are presented as mean ± S.D. (n = 3 independent biological replicates; ∗adjusted *p*-value < 0.05, ordinary one-way ANOVA followed by Sidak’s multiple comparisons test; adjusted *p*-value < 0.001 for EPHB2 siRNA plus control lentivirus *versus* control siRNA plus control lentivirus, adjusted *p*-value = 0.001 for EPHB2 siRNA plus FOSL1-overexpressing lentivirus *versus* control siRNA plus control lentivirus, and adjusted *p*-value < 0.001 for EPHB2 siRNA plus FOSL1-overexpressing lentivirus *versus* EPHB2 siRNA plus control lentivirus). *D*, representative transwell migration images of Schwann cells co-transfected with control siRNA plus control lentivirus, EPHB2 siRNA plus control lentivirus, or EPHB2 siRNA plus FOSL1-overexpressing lentivirus. Scale bar: 100 μm. *E*, quantification of the migration ability of transfected Schwann cells. Summarized data are presented as mean ± S.D. (n = 3 independent biological replicates; ∗adjusted *p*-value < 0.05, ordinary one-way ANOVA followed by Sidak’s multiple comparisons test; adjusted *p*-value < 0.001 for EPHB2 siRNA plus control lentivirus *versus* control siRNA plus control lentivirus, adjusted *p*-value = 0.012 for EPHB2 siRNA plus FOSL1-overexpressing lentivirus *versus* control siRNA plus control lentivirus, and adjusted *p*-value = 0.011 for EPHB2 siRNA plus FOSL1-overexpressing lentivirus *versus* EPHB2 siRNA plus control lentivirus). *F*, representative wound healing images of Schwann cells co-transfected with control siRNA plus control lentivirus, EPHB2 siRNA plus control lentivirus, or EPHB2 siRNA plus FOSL1-overexpressing lentivirus. Insert positions are labeled with *dashed lines*. Scale bar: 200 μm. *G*, quantification of relative cleaned area at 10 h after insert removal in transfected Schwann cells. Summarized data are presented as mean ± SD (n = 3 independent biological replicates; ∗adjusted *p*-value < 0.05, ordinary one-way ANOVA followed by Sidak’s multiple comparisons test; adjusted *p*-value = 0.009 for EPHB2 siRNA plus control lentivirus *versus* control siRNA plus control lentivirus, adjusted *p*-value = 0.117 for EPHB2 siRNA plus FOSL1-overexpressing lentivirus *versus* control siRNA plus control lentivirus, and adjusted *p*-value = 0.197 for EPHB2 siRNA plus FOSL1-overexpressing lentivirus *versus* EPHB2 siRNA plus control lentivirus).

Schwann cells were subsequently co-treated with EPHB2 siRNA and FOSL1-overexpressing lentivirus to determine whether overexpressed FOSL1 would rescue the inhibiting role of EPHB2 siRNA. CCK-8 assay showed that overexpressed FOSL1 in EPHB2 siRNA-transfected Schwann cells by lentivirus infection slightly increased the optical density values of Schwann cells from about 83.82% of the control group to about 90.77% (Fig. 8*A*). FOSL1 overexpression-induced changes in Schwann cell proliferation and migration were more robust. EdU proliferation assay showed that FOSL1-overexpressing lentivirus infection recovered the relative proliferation rate from about 57.19% of the control group in the EPHB2 siRNA transfected group to about 80.69% (Fig. 8, *B* and *C*). Transwell migration assay demonstrated that EPHB2 siRNA-suppressed Schwann cell migration was rescued by FOSL1-overexpressing lentivirus infection (Fig. 8, *D* and *E*). Similarly, the relative clean area decreased from about 1.53-fold of the control group in Schwann cells co-treated with EPHB-siRNA and control lentivirus to less than 1.30-fold of the control group in Schwann cells co-treated with EPHB-siRNA and FOSL1-overexpressing lentivirus (Fig. 8, *F* and *G*), indicating that FOSL1 overexpression abolishes EPHB2 siRNA-induced impairment of Schwann cell migration. The rescue effects of overexpressed FOSL1 on silenced EPHB2 fully support that FOSL1 binds to EPHB2, positively regulates EPHB2, and thus mediates Schwann cell proliferation and migration.

## Discussion

Schwann cells, different from many other differentiated cells, obtain extensive reprogramming capacity in response to peripheral nerve injury (19). Impaired Schwann cell plasticity hinders the functional recovery of injured peripheral nerves, indicating the importance of Schwann cell phenotype modulation in successful nerve regeneration (20).

Here, we examined the abundances of FOSL1 at the injured sites at 1, 4, 7, and 14 days after nerve injury and found that FOSL1 gene expression was obviously increased within the first 24 h as compared with the uninjured 0-day control. Compared with the immediate changes of FOSL1 mRNA, the increase in FOSL1 protein expression seemed to be lagged and FOSL1 protein expression kept increasing at relatively later time points. Considering that our data showed that up-regulated FOSL1 supports the migration of Schwann cells, it is likely that Schwann cells with elevated FOSL1 expression migrate toward the injured sites and contribute to further increased FOSL1 protein abundances.

FOSL1 has been identified as an inducing factor for the reprogramming of many different cell types, including melanocytes (21), glioblastoma cells (22), and Th17 cells (23). In the current study, we explored the effects of FOSL1 on Schwann cells by transfecting Schwann cells with siRNA segment against FOSL1 or infecting Schwann cells with FOSL1-overexpressing lentivirus and revealed that FOSL1 positively regulates the proliferation rate and migration ability of Schwann cells. *In vivo* examinations further supported that silencing FOSL1 decreases the enlargement of Schwann cell population and hinders the migration of Schwann cells from proximal and distal nerve segments towards the injured sites, indicating that FOSL1 may be an effective inducer of the reversion of mature Schwann cells to repair Schwann cells. In addition to the observed functional roles of FOSL1 in Schwann cells, it has been reported that FOSL1 is capable of facilitating the migration of endothelial cells as well as the assembly of endothelial cells into vessel structures (24, 25). Therefore, FOSL1 silencing may lead to a disadvantageous microenvironment by impairing Schwann cell plasticity and angiogenesis. Direct observations from SCG10 immunostaining of injured nerves and transmission electron microscope of myelin sheathes revealed that reduced FOSL1 induces retardatory axon regrowth and myelin sheath enwrapping following peripheral nerve injury. Moreover, functional investigations demonstrated that reduced FOSL1 leads to impaired motor function recovery and nerve conduction, suggesting that FOSL1 influences functional recovery of injured nerves by changing Schwann cell phenotype and altering the wound microenvironment.

We next investigated the mechanisms underlying FOSL1-regulated Schwann cell behavior by ChIP sequencing and RNA sequencing. Together with the top enrichment of signaling by receptor tyrosine kinase by Reactome signaling, EPHB2, a gene that encodes tyrosine-protein kinase receptor family member EPH-3, was found to be a potential downstream target of FOSL1 by ChIP-sequencing. EPHB2 expression was further identified to be down-regulated in Schwann cells transfected with FOSL1 siRNA by RNA sequencing and RT-PCR validation. In addition to the positive regulation of FOSL1 on EPHB2, the dynamic changes of FOSL1 and EPHB2 following peripheral nerve injury were also comparable.

Outcomes from the luciferase assay and the functional assessment of EPHB2 on Schwann cell behavior fully indicated that FOSL1 regulates Schwann cell behavior through its target gene EPHB2. EPHB2 signaling has been identified to be a major mediator of the sorting and organized migration of Schwann cells and the formation of Schwann cell cord that directs axon regrowth (26). It has been demonstrated that ephrin-B on fibroblasts activates EPHB2 receptors on Schwann cells and thus mediates Schwann cell sorting in a Sox2-dependent manner (26). Our current study implied that besides fibroblast-mediated changes, injury responses may directly regulate the abundance of EPHB2 in Schwann cells *via* elevating FOSL1 expression. Our study demonstrated that silencing EPHB2 hinders the migration ability of Schwann cells, indicating that EPHB2 may not only affect the movement direction but also regulate the movement speed of Schwann cells. The expression of Sox2 in Schwann cells, however, is not significantly altered after FOSL1 knockdown or overexpression according to our sequencing data, proposing that FOSL1 may not regulate the movement speed of Schwann cells *via* the stemness factor Sox2. In addition to cellular movement, our data indicate that similar to cutaneous squamous cell carcinoma cells (27), cervical cancer cells (28), and somatic stem cells (29), EPHB2 enhances the proliferation rate of Schwann cells, supplying new knowledge about the biological activity of EPHB2.

It is worth noting that in our current study, we used bulk sequencing data for the identification of dynamic changes of FOSL1 and its potential targets. The application of bulk sequencing may mask precise information about diverse cell types in peripheral nerves, including Schwann cells. A recent publication purified Schwann cells from the naïve and injured sciatic nerve and determined genetic changes in Schwann cells at 3, 5, and 7 days after nerve injury (30). FOSL1 expression in Schwann cells is noticeably up-regulated at multiple time points post-injury according to their sequencing data. EPHB2 expression also seems to be increased, especially at 5 days after nerve injury. Different from bulk sequencing data, FTH1 and TMEM231, two other genes whose expressions are significantly reduced in FOSL1 siRNA-transfected Schwann cells, are up-regulated in Schwann cells in the injured nerves (30). The inconsistency of gene expression levels in whole nerve tissues and purified Schwann cells indicates that it is helpful to apply single-cell sequencing to identify specific cellular and molecular changes during nerve regeneration. We preliminarily examined the biological function of FTH1 on Schwann cells and found that, different from FOSL1 knockdown, FTH1 knockdown leads to elevated Schwann cell proliferation and migration (data not shown). These results ruled out the possibility that FOSL1 regulates Schwann cell activity *via* targeting FTH1. Still, whether TMEM231 is the downstream target of FOSL1 is undetermined.

Besides the 0 to 3 kb promoter region investigated in our current study, FOSL1 may bind to other regions, for instance, the distal intergenic region. The upset plot showed that among the 47 FOSL1 bound elements in the distal intergenic region, a total of 3 genes, including SLC1A3, PIF1, and TMEM86A, were significantly differentially expressed after FOSL1 siRNA transfection. PIF1 and TMEM86A were upregulated in Schwann cells in the injured nerves according to the sequencing data of purified Schwann cells (30) and thus FOSL1 may bind to PIF1 and/or TMEM86A and regulate their expressions. In addition, using a less strict criterion to screen differentially expressed genes may help to identify more potential downstream targets of FOSL1.

AP-1 family member c-Jun has been widely cognized as a key regulator of Schwann cell phenotype. c-Jun controls the molecular changes of Schwann cells, mediates the generation of repair Schwann cells, and plays essential roles in peripheral nerve regeneration (5). High throughput analysis of gene expressions in the sciatic nerves of c-Jun mutant mice and their control littermates after nerve injury has identified a total of 172 genes with changed expressions in control and c-Jun mutants (14). However, by screening the abundances of these genes in Schwann cells transfected with FOSL1 siRNA or control siRNA, we found that only a small proportion of these genes were altered after FOSL1 knockdown. Thus, it is likely that c-Jun and FOSL1 may not come with a high degree of consistency for their target genes. Still, some regeneration and trophic support-related genes that were reduced in c-Jun mutants were also down-regulated after FOSL1 siRNA transfection. For instance, ARTN expression was significantly suppressed in Schwann cells transfected with FOSL1 siRNA. BDNF, GDNF, and GAP43, although were not significantly differentially expressed, showed a reduced expression trend in FOSL1 siRNA-transfected Schwann cells.

Collectively, our current study discovers FOSL1 as a transcription factor dysregulated at early time points after nerve injury, identify the valuable effects and underlying mechanisms of FOSL1 on Schwann cell plasticity and axon elongation, demonstrate the prospective roles of FOSL1 in neural remodeling and regeneration, and deepen our understanding of the importance of the AP-1 transcription factor family in microenvironment rebuilding and regenerative medicine.

## Experimental procedures

### Animals

Specific pathogen free (SPF) degree Sprague-Dawley (SD) rats were obtained from the Experimental Animal Center of Nantong University. Animal experiments were approved ethically by the Administration Committee of Experimental Animals, Jiangsu, China, and conducted according to the Institutional Animal Care Guidelines of Nantong University.

### Sciatic nerve crush and sample collection

Adult male SD rats (180–220 g) under anaesthesia were subjected to sciatic nerve crush injury as previously described with modifications (17). Briefly, a small incision was made in the left hind limb at the mid-thigh level and exposed sciatic nerves were crushed using hemostatic forceps for 30 s (twice with a period of 15 s for each time). Rat sciatic nerves at injured sites were collected at 1, 4, 7, and 14 days after surgery. Rats in the control group were sham-operated and designated as 0-day control.

### Real-time RT-PCR

Total RNA was extracted from rat sciatic nerves or cultured cells using RNA-Quick Purification Kit (Esunbio) and reverse transcribed to cDNA using HiScript II Q RT SuperMix for qPCR (+gDNA wiper) (Vazyme). Quantitative real-time RT-PCR was performed with ChamQ SYBR qPCR Master Mix (Vazyme) on an ABI StepOne System (Applied Biosystems). Primers were synthesized by Sangon Biotech and detailed information on primers are listed in Table S1. The relative expression levels of target genes were calculated using the comparative 2^−ΔΔCt^ method and normalized with reference gene GAPDH.

### Immunohistochemistry

Rat sciatic nerves were fixed with 4% PFA, dehydrated in 30% sucrose solution, and cut into 20 μm thick sections. The cyto-sections of sciatic nerves were incubated with primary antibodies anti-FOSL1 (1:500; sc-28310, Santa Cruz Biotechnology), anti-S100β (1:400; ab52642, Abcam), and anti-SCG10 (1:400, NBP1-49461, Novus Biologicals) at 4 °C overnight followed by secondary antibodies donkey anti-mouse-488 IgG (1:500; SA00013-5, Proteintech) and goat anti-rabbit-Cy3 IgG (1:500; SA00009-2; Proteintech) for 2 h at room temperature. Nucleus was stained with DAPI Fluoromount-G (SouthernBiotech). Images were taken under a Zeiss Axio Imager M2 microscope (Carl Zeiss Microscopy GmbH) and quantifications were performed using Image J.

### Schwann cell culture and treatment

Schwann cells were isolated from neonatal rat sciatic nerves by digesting nerve segments with collagenase Type I (Sigma) and Trypsin (Sigma), incubating with a complete medium containing DMEM (Corning), 10% FBS (Gibco), and 1% penicillin-streptomycin (ScienCell), and removing contaminating fibroblasts with anti-Thy1.1 (Sigma) and rabbit complement (Invitrogen). Purified Schwann cells were maintained in complete medium supplied with 2 μM forskolin (Sigma) and 10 ng/ml HRG (R&D Systems Inc) until confluence at 37 °C in 5% CO_2_.

Cultured Schwann cells were transfected with FOSL1 siRNA (target sequence: TCCCAGAAGAAGACAAGAA), EPHB2 siRNA (target sequence: CAACGCTGAAGAAGTGGAT) or control siRNA (random sequence; RibiBio) using Lipofectamine RNAiMAX (Invitrogen) to knockdown FOSL1 or EPHB2 expression. Schwann cells were infected with FOSL1-overexpressing lentivirus (pRlenti-SFH-EGFP-P2A-Puro-CMV-FOSL1-3Flag-2) or control lentivirus (GL120, pRLenti-SFH-EGFP-P2A-Puro-CMV-3FLAG; OBiO) to increase FOSL1 expression.

### CCK-8 assay

Schwann cells were suspended in 100 μl complete medium and seeded onto 96-well culture plates at a density of 2 × 10^5^ cells/ml. After 24-h culture, Schwann cells were treated with CCK-8 solution (Cell Counting Kit-8, Beyotime) for 2 h according to the manufacturer’s instructions. The optical density value was measured at 450 nm using a microplate reader (Bio-Rad).

### EdU proliferation assay

Schwann cells suspended in 100 μl complete medium were seeded onto 96-well culture plates at a density of 2 × 10^5^ cells/ml, treated with 50 μM EdU using EdU Alexa Fluro 647 Imaging Kit (Invitrogen), and fixed with 4% PFA. Fixed cells were stained with Apollo dye solution and Hoechst 33,342 (Ribibio) to label proliferating cells and total cells, respectively. Cell proliferation rate was determined according to the percentage of EdU-labeled proliferating cells in total cells. Images were taken under a DMR fluorescence microscope (Leica Model DMi8, Leica Microsystems CMS GmbH) and quantified using Image-Pro Plus (Media Cybernetics).

### Transwell migration assay

Schwann cells suspended in 100 μl DMEM were seeded onto fibronectin (Millipore)-coated top chamber of a 6.5 mm transwell with 8 μm pores (Corning) at a density of 4 × 10^5^ cells/ml. Schwann cells were allowed to migrate towards the lower chamber filled with 600 μl complete medium for 24 h at 37 °C in 5% CO_2_. After wiping Schwann cells left on the upper surface of the top chamber with a cotton swab, Schwann cells adhering to the bottom surface were fixed with 4% PFA, stained with crystal violet (Solarbio), and observed under a DMR inverted microscope (Leica Model DMI3000B, Leica Microsystems). Crystal violet was dissolved using 33% acetic acid (Xilong Scientific) and the optical density value was measured at 570 nm using a Synergy 2 Multi-Mode Microplate Reader (BioTek).

### Wound healing assay

Schwann cells suspended in 80 μl complete medium were seeded onto wound healing culture inserts (ibidi) placed on 6-well plates at a density of 2 × 10^5^ cells/ml. Wound healing culture inserts were removed after cell confluence and culture medium was changed to DMEM containing 0.5% FBS and 0.15 μg/ml mitocycin C (Sigma). Images were taken at 0 and 10 h after insert removal using a DMR inverted microscope (Leica Microsystems). Cleaned areas were quantified using Image-Pro Plus (Media Cybernetics).

### *In vivo* examination

*In vivo* FOSL1 siRNA (FOSL1-siRNA, target sequence: TCCCAGAAGAAGACAAGAA and FOSL1-siRNA-2, target sequence: CATCGAAAGAGTAGCAGCA; RibiBio) or control siRNA (RibiBio) was mixed with equal volume of Matrigel (Corning) and injected to the injured sites of rat sciatic nerves immediately after nerve crush injury. At 1 and 4 days post surgery, rat sciatic nerves were harvested, fixed with 4% PFA, and immunostained to observe Schwann cell migration and axon elongation. EdU (Invitrogen) was injected intraperitoneally at 24 h prior to nerve tissue collection to determine cell proliferation. At 1, 2, and 3 weeks post surgery, gait analysis was performed by placing rats in an enclosed walkway and recording their footprints using the CatWalk XT system (Noldus Information Technology, Wageningen, Netherlands). SFI was calculated based on paw length and toe spread according to the formula SFI = −38.3 [(EPL − NPL)/NPL] + 109.5 [(ETS − NTS)/NTS] + 13.3 [(EIT − NIT)/NIT] − 8.8, with EPL indicating experimental paw length, NPL indicating normal paw length, ETS indicating experimental toe spread, NTS indicating normal toe spread, EIT indicating experimental intermediary toe spread, and NIT indicating normal intermediary toe spread. At 2 weeks post surgery, rat sciatic nerves were fixed with 4% glutaraldehyde and embedded in Epon 812 epoxy resin (Sigma). Nerve sections collected at the same distance distal to the injured site were stained with lead citrate and uranyl acetate and observed using transmission electron microscope (JEOL Ltd) and quantified using Image J. At 3 weeks post surgery, electrophysiological parameters were measured by inserting electrodes into proximal nerve segments, distal nerve segments, and the mid-belly of gastrocnemius and applying a 5 mA electrical stimulus using Keypoint 2 portable electromyography (Dantec).

### ChIP sequencing and RNA sequencing

Schwann cells infected with FOSL1-overexpressing lentivirus were cross-linked in 1% PFA, quenched with 125 mM glycine, lysed by micrococcal nucleic acid enzymatic hydrolysis, and sonicated to obtain DNA fragments. ChIP sequencing and library preparation were performed by Genechem Co, LTD. Peaks calling and raw signal tracks in comparison to input controls were visualized by IGV genome viewer tool. ChIP sequencing data were stored in GEO database with accession number GSE214449.

Schwann cells infected with FOSL1 siRNA or control siRNA were subjected to RNA isolating and transcriptome sequencing. RNA sequencing was performed on the Illumina sequencing platform by Genedenovo Biotechnology Co, Ltd. Gene abundances were quantified using the FPKM formula. Genes with a fold change >2 or < −2 and a false discover rate <0.05 were considered as significantly differently expressed. RNA sequencing data were stored in SRA database with accession number PRJNA614580.

### Luciferase assay

HEK 293T cells were seeded onto 24-well plates at a density of 4 × 10^5^ cells/well and co-transfected with wild-type or mutant EPHB2 promoter reporter vector and FOSL1 or empty vector (GeneChem). For EPHB2 promoter reporter vector, wild-type sequence or mutant sequence (deletion mutation from −152 bp to −141 bp) was constructed into the pGL3-basic vector. For FOSL1 vector, the CDS sequence of FOSL1 was constructed into the pcDNA3.1 vector. The relative luciferase activity was determined using Dual-luciferase Reporter Assay System (Invitrogen) according to the manufacturer’s instructions and normalized with Renilla luciferase reporter.

### Statistical analysis

Numerical data were displayed as mean ± SD values. Statistics was analyzed by unpaired Student’s *t* test or ordinary one-way ANOVA followed by Dunnett’s multiple comparisons test or Sidak’s multiple comparisons test using GraphPad Prism (GraphPad software). A *p*-value less than 0.05 was considered statistically significant. Details about statistical tests were presented in figure legends.

## Data availability

Data are available from the corresponding author on reasonable request. ChIP sequencing data were stored in GEO database with accession number GSE214449. RNA sequencing data were stored in SRA database with accession number PRJNA614580.

## Supporting information

This article contains supporting information.

## Conflict of interest

The authors declare no competing interests.

## References

[1] Jones S., Eisenberg H.M., Jia X. (2016). Advances and future applications of augmented peripheral nerve regeneration. Int. J. Mol. Sci..

[2] Yi S., Zhang Y., Gu X., Huang L., Zhang K., Qian T. (2020). Application of stem cells in peripheral nerve regeneration. Burns Trauma.

[3] Stierli S., Imperatore V., Lloyd A.C. (2019). Schwann cell plasticity-roles in tissue homeostasis, regeneration, and disease. Glia.

[4] Babetto E., Wong K.M., Beirowski B. (2020). A glycolytic shift in Schwann cells supports injured axons. Nat. Neurosci..

[5] Jessen K.R., Mirsky R. (2016). The repair Schwann cell and its function in regenerating nerves. J. Physiol..

[6] Clements M.P., Byrne E., Camarillo Guerrero L.F., Cattin A.L., Zakka L., Ashraf A. (2017). The wound microenvironment reprograms Schwann cells to invasive mesenchymal-like cells to drive peripheral nerve regeneration. Neuron.

[7] Jessen K.R., Arthur-Farraj P. (2019). Repair Schwann cell update: adaptive reprogramming, EMT, and stemness in regenerating nerves. Glia.

[8] Vaquerizas J.M., Kummerfeld S.K., Teichmann S.A., Luscombe N.M. (2009). A census of human transcription factors: function, expression and evolution. Nat. Rev. Genet..

[9] Lambert S.A., Jolma A., Campitelli L.F., Das P.K., Yin Y., Albu M. (2018). The human transcription factors. Cell.

[10] Takahashi K., Yamanaka S. (2006). Induction of pluripotent stem cells from mouse embryonic and adult fibroblast cultures by defined factors. Cell.

[11] Takahashi K., Tanabe K., Ohnuki M., Narita M., Ichisaka T., Tomoda K. (2007). Induction of pluripotent stem cells from adult human fibroblasts by defined factors. Cell.

[12] Takahashi K., Yamanaka S. (2016). A decade of transcription factor-mediated reprogramming to pluripotency. Nat. Rev. Mol. Cell Biol..

[13] Nocera G., Jacob C. (2020). Mechanisms of Schwann cell plasticity involved in peripheral nerve repair after injury. Cell Mol. Life Sci..

[14] Arthur-Farraj P.J., Latouche M., Wilton D.K., Quintes S., Chabrol E., Banerjee A. (2012). c-Jun reprograms Schwann cells of injured nerves to generate a repair cell essential for regeneration. Neuron.

[15] Yi S., Tang X., Yu J., Liu J., Ding F., Gu X. (2017). Microarray and qPCR analyses of Wallerian degeneration in rat sciatic nerves. Front. Cell Neurosci..

[16] Lykke-Andersen S., Zumer K., Molska E.S., Rouviere J.O., Wu G., Demel C. (2021). Integrator is a genome-wide attenuator of non-productive transcription. Mol. Cell.

[17] Yi S., Zhang H., Gong L., Wu J., Zha G., Zhou S. (2015). Deep sequencing and bioinformatic analysis of lesioned sciatic nerves after crush injury. PLoS One.

[18] Zhao L., Yi S. (2019). Transcriptional landscape of alternative splicing during peripheral nerve injury. J. Cell. Physiol..

[19] Merrell A.J., Stanger B.Z. (2016). Adult cell plasticity *in vivo*: de-differentiation and transdifferentiation are back in style. Nat. Rev. Mol. Cell Biol..

[20] Painter M.W., Brosius Lutz A., Cheng Y.C., Latremoliere A., Duong K., Miller C.M. (2014). Diminished Schwann cell repair responses underlie age-associated impaired axonal regeneration. Neuron.

[21] Maurus K., Hufnagel A., Geiger F., Graf S., Berking C., Heinemann A. (2017). The AP-1 transcription factor FOSL1 causes melanocyte reprogramming and transformation. Oncogene.

[22] Pecce V., Verrienti A., Fiscon G., Sponziello M., Conte F., Abballe L. (2021). The role of FOSL1 in stem-like cell reprogramming processes. Sci. Rep..

[23] Shetty A., Tripathi S.K., Junttila S., Buchacher T., Biradar R., Bhosale S.D. (2022). A systematic comparison of FOSL1, FOSL2 and BATF-mediated transcriptional regulation during early human Th17 differentiation. Nucleic Acids Res..

[24] Evellin S., Galvagni F., Zippo A., Neri F., Orlandini M., Incarnato D. (2013). FOSL1 controls the assembly of endothelial cells into capillary tubes by direct repression of alphav and beta3 integrin transcription. Mol. Cell Biol..

[25] Galvagni F., Orlandini M., Oliviero S. (2013). Role of the AP-1 transcription factor FOSL1 in endothelial cells adhesion and migration. Cell Adh. Migr..

[26] Parrinello S., Napoli I., Ribeiro S., Wingfield Digby P., Fedorova M., Parkinson D.B. (2010). EphB signaling directs peripheral nerve regeneration through Sox2-dependent Schwann cell sorting. Cell.

[27] Farshchian M., Nissinen L., Siljamaki E., Riihila P., Toriseva M., Kivisaari A. (2015). EphB2 promotes progression of cutaneous squamous cell carcinoma. J. Invest Dermatol..

[28] Duan S., Wu A., Chen Z., Yang Y., Liu L., Shu Q. (2018). miR-204 regulates cell proliferation and invasion by targeting EphB2 in human cervical cancer. Oncol. Res..

[29] N'Tumba-Byn T., Yamada M., Seandel M. (2020). Loss of tyrosine kinase receptor Ephb2 impairs proliferation and stem cell activity of spermatogonia in culturedagger. Biol. Reprod..

[30] Brosius Lutz A., Lucas T.A., Carson G.A., Caneda C., Zhou L., Barres B.A. (2022). An RNA-sequencing transcriptome of the rodent Schwann cell response to peripheral nerve injury. J. Neuroinflammation.

