# Supplementary material for: FOSL1 modulates Schwann cell responses in the wound microenvironment and regulates peripheral nerve regeneration
Source: J Biol Chem. 2023 Nov 8;299(12):105444. doi: 10.1016/j.jbc.2023.105444 (PMC10716580; doi:10.1016/j.jbc.2023.105444)
Supplement: Supplemental Table S1 [file mmc1.doc]

**Table S1. List of primer sequences.** Gene symbol, primer sequence, and amplicon size are provided.

| **Gene** | **Fragment** | **Sequence (5'-3')** | **Product** |
| --- | --- | --- | --- |
| **FOSL1** | FOSL1-Forward | GTGAGACGAGAGCGGAACAA | 158 bp |
|  | FOSL1-Reverse | TCAAGGCGTTCCTTCTGCTT |  |
| **ADAM10** | ADAM10-Forward | TCCTGCCGTTTCACTCTGTC | 153 bp |
|  | ADAM10-Reverse | CTGAATGTGCCCGAGTTCCT |  |
| **EPHB2** | EPHB2-Forward | TAACAGACGGGGGTTTGAGC | 207 bp |
|  | EPHB2-Reverse | TCACCAAATTCCCCTGCTCC |  |
| **FTH1** | FTH1-Forward | GCCAGAACTACCACCAGGAC | 218 bp |
|  | FTH1-Reverse | GAAGATTCGTCCACCTCGCT |  |
| **SLC39A10** | SLC39A10-Forward | ATTCTGGCCGTTCAAGAGGG | 179 bp |
|  | SLC39A10-Reverse | TCGTGCGGGTGTTTATCCTC |  |
| **TMEM231** | TMEM231-Forward | ACGTACCCCACTTTCAACCG | 165 bp |
|  | TMEM231-Reverse | CAGGATAAGCTGCACACCGA |  |
| **NR4A3** | NR4A3-Forward | TGGTGAAGGAAGTTGTGCGT | 202 bp |
|  | NR4A3-Reverse | TGGTCGGTGGGACAGTATCT |  |
| **NUDT16L1** | NUDT16L1-Forward | CGTGTCCCACTGTACACACA | 176 bp |
|  | NUDT16L1-Reverse | GCCTTCTTCTGCTTCTCCGT |  |
| **GAPDH** | GAPDH-Forward | ACAGCAACAGGGTGGTGGAC | 252 bp |
|  | GAPDH-Reverse | TTTGAGGGTGCAGCGAACTT |  |
